# Genomes of cressdnaviruses, microviruses, a gyrovirus, and a caudovirus identified in the fecal samples of a mallard and double-crested cormorant

**DOI:** 10.1128/mra.00332-24

**Published:** 2024-05-29

**Authors:** Anthony Khalifeh, Shawnpreet Sahnan, Shubham Kale, Diego Olivo, Michael C. Lund, Simona Kraberger, Arvind Varsani

**Affiliations:** 1 The Biodesign Center for Fundamental and Applied Microbiomics, Center for Evolution and Medicine, School of Life Sciences, Arizona State University, Tempe, Arizona, USA; 2 Center for Research in Engineering, Science and Technology, Paradise Valley High School, Phoenix, Arizona, USA; 3 Structural Biology Research Unit, Department of Integrative Biomedical Sciences, University of Cape Town, Rondebosch, Cape Town, South Africa; Portland State University, Portland, Oregon, USA

**Keywords:** gyrovirus, microvirus, caudovirus, mallard, double-crested cormorant

## Abstract

Mallards and double-crested cormorants have a broad distribution across North America. In the fecal sample from two individual mallard and double-crested cormorant, we determined the genomes of a caudovirus, microviruses (*n* = 6), cressdnaviruses (*n* = 35), and a gyrovirus (chicken anemia virus, CAV). Here, we report double-crested cormorant as a CAV host.

## ANNOUNCEMENT

Mallards (*Anas platyrhynchos*) and double-crested cormorants (*Nannopterum auritum*) are widely distributed across North America. Although viruses in 23 families have been identified as circulating in mallards, relatively less is known about those in double-crested cormorants (1). To date, only viruses in the *Flaviviridae*, *Orthomyxoviridae,* and *Paramyxoviridae* have been reported in double-crested cormorants (1).

**Fig 1:**
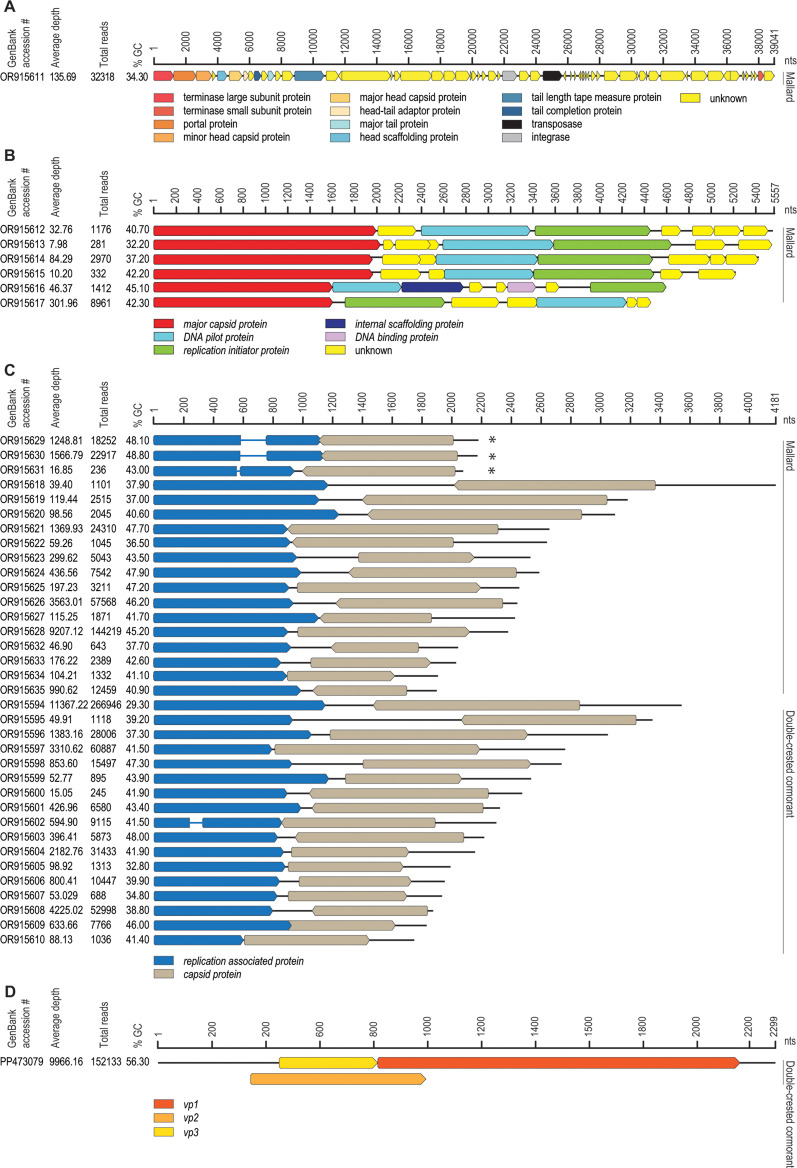
Summary of the accession number, read depth, number of reads mapped to the genome, percentage of GC content, and genome organization of the viruses identified from the mallard and double-crested cormorant fecal samples. (**A**) Unclassified caudovirus (*Duplodnaviria; Heunggongvirae; Uroviricota; Caudoviricetes*). (**B**) Unclassified microviruses (*Monodnaviria; Sangervirae; Phixviricota; Malgrandaviricetes; Petitvirales; Microviridae*). (**C**) Unclassified genomoviruses in the phylum *Cressdnaviricota* (*Repensiviricetes; Geplafuvirales; Genomoviridae*) (marked with an asterisk) and all unclassified cressdnaviruses. (**D**) Chicken anemia virus (*Anelloviridae; Gyrovirus; Gyrovirus chickenanemia*).

As part of our effort to identify viruses in migratory birds that concentrate around bodies of freshwater (2, 3) in the southwestern USA (part of the Pacific Flyway for migratory birds), we collected two fecal samples, one from a mallard and one from a double-crested cormorant at Kiwanis Park, Tempe (AZ, USA) on 12 January 2021. The samples were collected in 2 mL tubes following a visual defecation observation. The fecal samples were homogenized in 2 mL of SM buffer, centrifuged at 10,000 × *g* for 10 min, and the supernatant from each sample was filtered through 0.45 and 0.2 µm syringe filters. Viral DNA was extracted from 200 µL of the filtrate with the High Pure Viral Nucleic Acid Kit (Roche, USA). Circular viral DNA was enriched using rolling circle amplification (RCA) with the Templiphi 100 Amplification Kit (GE Healthcare, USA). Illumina sequencing libraries were generated from the RCA products using the DNA TrueSeq Nano Kit and sequenced on an Illumina HiSeq4000. Trimmomatic v0.39 (4) was used to trim the raw paired-end reads (2 × 150 nt) and were then *de novo* assembled using MEGAHIT v1.2.9 (5). Contigs > 1,000 nt in length were analyzed using BLASTx (6) against a viral RefSeq protein sequence database (release 207) to identify viral-like sequences. The viral-like sequences were checked for terminal redundancy to identify circular genomes. All contigs were annotated with Cenote-taker 2 (7) with manual checks to identify spliced coding regions (Fig. 1). Read depth for viral contigs was determined using BBMap (8). All bioinformatic tools were run with default parameters.

In the mallard sample, we identified a caudovirus, 6 microviruses, 3 genomoviruses, and 15 unclassified cressdnaviruses (Fig. 1). A gyrovirus and 17 cressdnaviruses (Fig. 1) were identified in the double-crested cormorant sample. A BLASTn (9) analysis of the virus genomes identified in this study against the non-redundant nucleotide database shows that 37 of the 43 genomes have low similarity (65.88%–89.09%) over a low genome coverage (3%–58%) with *E*-values ranging from 0 to 2 × 10^−5^ (Table 1). The viruses with accessions OR915625, OR915629, OR915630, and OR915634 from mallard and OR915600 and PP473079 from double-crested cormorant have >70% genome coverage with 73.77%–99.16% identity and *E*-values of 0 in the BLASTn analysis (Table 1). Other than chicken anemia virus (CAV), which infects avian species, we are unable to determine the true hosts of the caudovirus, microviruses, and cressdnaviruses. CAV has mainly been identified in chickens (*Gallus gallus*) where it has a negative impact on chicken health. Although CAV has been identified in two other avian hosts, including waterfowl (10) and neognath (11), here we report its identification in double-crested cormorant as a host.

**TABLE 1:** Summary of the top BLASTn hit to the genomes of the viruses identified in this study

		Top BLASTn hit				
Query accession	Query virus	Accession	Virus	Query cover (%)	*E* value	Identity (%)
OR915611	Malazfec virus 1 isolate K14_7	BK023036	Caudoviricetes sp. ct1fq4	13	0	70.66
OR915612	Malazfec virus 2 isolate K14_247	MH617122	Microviridae sp. ctcf586	11	1.00E-45	67.32
OR915613	Malazfec virus 3 isolate K14_248	MH617122	Microviridae sp. ctcf586	39	2.00E-143	73.31
OR915614	Malazfec virus 4 isolate K14_261	MH617096	Microviridae sp. ctch906	32	2.00E-91	66.59
OR915615	Malazfec virus 5 isolate K14_287	MT310011	Microvirus sp. BS1_235	55	1.00E-158	70.48
OR915616	Malazfec virus 6 isolate K14_352	MH992207	Apis mellifera associated microvirus 25 INH_SP_247	28	1.00E-63	68.18
OR915617	Malazfec virus 7 isolate K14_385	OP549816	Wigfec virus K19_181	3	2.00E-16	72.99
OR915618	Malazfec virus 8 isolate K14_433	MT201704	Circoviridae sp. CN13_L15_1359	19	2.00E-92	69.93
OR915619	Malazfec virus 9 isolate K14_711	MT263553	Crucivirus-133 BS_350	20	3.00E-68	69.70
OR915620	Malazfec virus 10 isolate K14_738	KY487859	Uncultured virus clone CG190	23	1.00E-79	69.74
OR915621	Malazfec virus 11 isolate K14_980	MT206330	Circoviridae sp. CN3_L17_509	9	2.00E-32	73.88
OR915622	Malazfec virus 12 isolate K14_992	MT202512	Circoviridae sp. CN13_L15_6418	26	3.00E-36	65.88
OR915623	Malazfec virus 13 isolate K14_1076	MT204432	Circoviridae sp. CN3_L16_1831	7	2.00E-30	77.25
OR915624	Malazfec virus 14 isolate K14_1094	KM598391	Odonata-associated circular virus-8 OdasCV-8-US-1739LM1-12	40	3.00E-118	69.52
OR915625	Malazfec virus 15 isolate K14_1126	MW697512	Arizlama virus AZLM_773	99	0	75.04
OR915626	Malazfec virus 16 isolate K14_1141	KM598391	Odonata-associated circular virus-8 OdasCV-8-US-1739LM1-12	41	9.00E-93	68.03
OR915627	Malazfec virus 17 isolate K14_1154	MN891812	Kummerowia striata CRESS virus strain pt119-gem-2	40	0	74.90
OR915628	Malazfec virus 18 isolate K14_1189	KM598391	Odonata-associated circular virus-8 OdasCV-8-US-1739LM1-12	36	4.00E-122	71.71
OR915629	Malazfec virus 19 isolate K14_1348	MT309903	Genomoviridae sp. 6402_219	100	0	98.46
OR915630	Malazfec virus 20 isolate K14_1356	MW678977	Genomoviridae sp. D5_996	98	0	73.76
OR915631	Malazfec virus 21 isolate K14_1449	MT205580	Geminiviridae sp. CN11_L16_2058	9	6.00E-12	69.54
OR915632	Malazfec virus 22 isolate K14_1490	MN891811	Kummerowia striata CRESS virus strain pt119-gem-1	11	7.00E-17	69.39
OR915633	Malazfec virus 23 isolate K14_1509	KP153407	Lake Sarah-associated circular virus-9 LSaCV-9-LSSO-2013	52	0	89.09
OR915634	Malazfec virus 24 isolate K14_1646	MK570179	Capybara virus 17_cap1_330	70	0	73.77
OR915635	Malazfec virus 25 isolate K14_1670	KY487903	Uncultured virus CG234	16	5.00E-38	71.79
OR915594	Comazfec virus 1 isolate K8_174	OM154589	Cressdnaviricota sp. isolate Miresoil virus 238	17	4.00E-68	70.45
OR915595	Comazfec virus 2 isolate K8_190	BK029143	Cressdnaviricota sp. isolate ct0WU4, partial genome	5	8.00E-32	78.07
OR915596	Comazfec virus 3 isolate K8_233	MT208121	Nanoviridae sp. isolate CN8_L19_778	22	4.00E-54	68.37
OR915597	Comazfec virus 4 isolate K8_281	MT200973	Circoviridae sp. isolate CN23_L12_4	15	2.00E-50	72.41
OR915598	Comazfec virus 5 isolate K8_283	MW202424	CRESS virus sp. isolate ctY8t820	32	1.00E-104	70.63
OR915599	Comazfec virus 6 isolate K8_313	MW697508	Arizlama virus isolate AZLM_25863	58	0	74.83
OR915600	Comazfec virus 7 isolate K8_328	MT209245	Circoviridae sp. isolate CN13_L19_1194	82	0	80.55
OR915601	Comazfec virus 8 isolate K8_369	MW697480	Arizlama virus isolate AZLM_25635	25	4.00E-53	72.73
OR915602	Comazfec virus 9 isolate K8_376	OP549820	Wigfec virus K19_450	5	2.00E-06	73.44
OR915603	Comazfec virus 10 isolate K8_396	MT206121	Circoviridae sp. isolate CN23_L17_1863	4	3.00E-09	78.26
OR915604	Comazfec virus 11 isolate K8_410	MW697496	Arizlama virus isolate AZLM_853	3	2.00E-11	87.88
OR915605	Comazfec virus 12 isolate K8_466	MT205814	Circoviridae sp. isolate CN3_L17_1849	7	2.00E-12	73.20
OR915606	Comazfec virus 13 isolate K8_476	MT203839	Nanoviridae sp. isolate CN8_L16_3306	4	2.00E-05	76.92
OR915607	Comazfec virus 14 isolate K8_485	MH616992	Circoviridae sp. isolate ctcf147	23	6.00E-43	70.11
OR915608	Comazfec virus 15 isolate K8_502	MT204555	Circoviridae sp. isolate CN3_L16_2825	12	3.00E-21	72.15
OR915609	Comazfec virus 16 isolate K8_523	KY884302	Garrulus glandarius associated circular virus 1 strain GgaCV-1/1	34	3.00E-40	66.93
OR915610	Comazfec virus 17 isolate K8_553	MW237847	Chicken proventriculitis-associated circular virus 23 strain CPACV-23	20	6.00E-36	71.31
PP473079	Chicken anemia virus isolate K8_371	MW091352	Chicken anemia virus strain 18R014B	100	0	99.16

## Data Availability

The sequences have been deposited in NCBI databases under BioProject PRJNA1045671; BioSample SAMN38452337 and SAMN38452338; SRA SRR26982314 and SRR26982315. The annotated genomes have been deposited in GenBank under accession numbers OR915594-OR915635 and PP473079.

## References

[1] Hatcher EL, Zhdanov SA, Bao Y, Blinkova O, Nawrocki EP, Ostapchuck Y, Schäffer AA, Brister JR. 2017. Virus variation resource - improved response to emergent viral outbreaks. Nucleic Acids Res 45:D482–D490. doi:10.1093/nar/gkw1065 27899678 PMC5210549

[2] Khalifeh A, Custer JM, Kraberger S, Varsani A. 2021. Novel viruses belonging to the family Circoviridae identified in wild American wigeon samples. Arch Virol 166:3437–3441. doi:10.1007/s00705-021-05236-2 34542726

[3] Olivo D, Khalifeh A, Custer JM, Kraberger S, Varsani A. 2024. Diverse small circular DNA viruses identified in an American wigeon fecal sample. Microorganisms 12:196. doi:10.3390/microorganisms12010196 38258021 PMC10821283

[4] Bolger AM, Lohse M, Usadel B. 2014. Trimmomatic: a flexible trimmer for Illumina sequence data. Bioinformatics 30:2114–2120. doi:10.1093/bioinformatics/btu170 24695404 PMC4103590

[5] Li D, Luo R, Liu CM, Leung CM, Ting HF, Sadakane K, Yamashita H, Lam TW. 2016. MEGAHIT v1.0: a fast and scalable metagenome assembler driven by advanced methodologies and community practices. Methods 102:3–11. doi:10.1016/j.ymeth.2016.02.020 27012178

[6] Altschul SF, Gish W, Miller W, Myers EW, Lipman DJ. 1990. Basic local alignment search tool. J Mol Biol 215:403–410. doi:10.1016/S0022-2836(05)80360-2 2231712

[7] Tisza MJ, Belford AK, Domínguez-Huerta G, Bolduc B, Buck CB. 2021. Cenote-Taker 2 democratizes virus discovery and sequence annotation. Virus Evol 7:veaa100. doi:10.1093/ve/veaa100 33505708 PMC7816666

[8] Bushnell B. 2014. BBMap: a fast, accurate, splice-aware aligner. Berkeley, CA (United States). Lawrence Berkeley National Lab.(LBNL)

[9] Altschul SF, Gish W, Miller W, Myers EW, Lipman DJ. 1990. Basic local alignment search tool. J Mol Biol 215:403–410. doi:10.1016/S0022-2836(05)80360-2 2231712

[10] Pauly M, Snoeck CJ, Phoutana V, Keosengthong A, Sausy A, Khenkha L, Nouanthong P, Samountry B, Jutavijittum P, Vilivong K, Hübschen JM, Black AP, Pommasichan S, Muller CP. 2019. Cross-species transmission of poultry pathogens in backyard farms: ducks as carriers of chicken viruses. Avian Pathol 48:503–511. doi:10.1080/03079457.2019.1628919 31199168

[11] A Duarte M, F Silva JM, R Brito C, S Teixeira D, L Melo F, M Ribeiro B, Nagata T, Campos FS. 2019. Faecal virome analysis of wild animals from Brazil. Viruses 11:803. doi:10.3390/v11090803 31480274 PMC6784175

